# Pediatric head trauma algorithm for head CT decision-making in the emergency department

**DOI:** 10.1186/s44158-025-00238-x

**Published:** 2025-04-10

**Authors:** Gabriele Savioli, Iride Francesca Ceresa, Andrea Piccioni, Yaroslava Longhitano, Raymond Planinsic, Michele Dorfsman, Antonio Voza, Federica Manzoni, Giorgia Caputo, Abdelouahab Bellou, Luigi La Via, Christian Zanza

**Affiliations:** 1 Department of Emergency Medicine, IRCCS Polyclinic San Matteo, Pavia, Italia; 2 Department of Emergency Medicine, Public Hospital, Vigevano, Italia; 3 Department of Emergency Medicine, Foundation Agostino Gemelli Hospital, UCSC, Rome, Italy; 4 Department of Anesthesiology and Perioperative Medicine, University of Pittsburgh, Pittsburgh, PA USA; 5 Department of Emergency Medicine, University of Pittsburgh Medical Center, Pittsburgh, PA USA; 6 Department of Emergency Medicine, Emergency Medicine Residency Program-Humanitas University Hospital, Rozzano, Italy; 7 Health Promotion-Environmental Epidemiology Unit, Hygiene and Health Prevention Department, Health Protection Agency, Pavia, Italy; 8 Department of Anesthesia and Intensive Care, San Luigi Gonzaga Hospital, Turin, Orbassano Italy; 9 Institute of Sciences in Emergency Medicine, Department of Emergency Medicine, Guangdong Provincial People’s Hospital, Guangdong Academy of Medical Sciences, Guangzhou, China; 10 Department of Anesthesia and Intensive Care, University Hospital Policlinico “G. Rodolico–San Marco”, Catania, Italy; 11 Geriatric Medicine Residency Program, University of Rome “Tor Vergata”, Rome, Italy

**Keywords:** Pediatric head trauma, PECARN algorithm, Computed tomography, Emergency department crowding, Clinical decision rules, Neuroimaging protocols

## Abstract

**Background:**

Traumatic brain injury is a common cause of admission in Emergency Department (ED) for pediatric patients. The aim of this study was to evaluate the application of the Pediatric Emergency Care Applied Research Network (PECARN) traumatic brain injury (TBI) algorithm in ED for head CT decision-making in pediatric patients. The secondary objective was to evaluate the impact of adherence to this protocol on ED crowding, length of stay, and boarding time.

**Methods:**

We conducted a retrospective study including children aged ≤ 15 years who were admitted in a level 2 trauma center ED for mild TBI from 1 January 2016 to 31 December 2019. Collected data included amnesia, symptoms, demographics, outcomes, length of ED stay, the patient’s outcomes, including intracranial injuries (ICI) and injuries requiring neurosurgery.

**Results:**

A total of 1372 children with mild TBI were included. More than half of the patients were male (59.8%) and ≥ 2 years of age (63.2%). Most of the trauma events (58%) were caused by home injury. Neurosurgical consultation (59.4%) was the most common intervention in the ED. Only 4.3% of patients required neuroimaging and 7 children had intracranial hemorrhage, with only 1 requiring immediate neurosurgical intervention. There were no re-admissions for bleeding. The adoption of this protocol had no negative impact on crowding, and a reduction of ED length of stay.

**Conclusions:**

The adoption of the PECARNE algorithm led to fewer brain computed tomography scans with good clinical outcomes without increasing crowding.

## Introduction

Pediatric head trauma is a leading cause of death and disability in children under 15, affecting 250 per 100,000 people annually, with a mortality rate of 6.8% [1, 2]. Males face 1.5–2 times higher risk, and incidence rates are increasing [3]. In the USA, children represent one-third of the 1.7 million annual head injuries [2]. The nature and severity of brain injuries vary by age, with infants being particularly vulnerable to shaking injuries due to weak neck muscles [3–5]. Among adolescents, falls and road accidents account for 9% of fatalities [1]. While head trauma frequently leads to ED visits requiring CT imaging, 80% of cases are mild, with only 3% requiring hospitalization and intensive care [6]. The PECARN decision support tool helps identify traumatic brain injury (TBI) while potentially reducing unnecessary CT scans and detecting cases requiring neurosurgery [7–16]. Additionally, it may help decrease ED exposure time [4]. This study aimed to evaluate how the PECARN TBI algorithm affects CT utilization in pediatric ED patients with mild head trauma, while also assessing its impact on ED crowding, length-of-stay (LOS), and boarding.

## Material and methods

### Study design

This observational study was performed as a retrospective analysis of the electronic health records of consecutive pediatric patients admitted to the University Hospital “IRCCS Policlinico San Matteo”, a level 2 trauma center, located in Pavia (Italy) from January 1 st 2016, to December 31 th 2019. During this period, 1892 pediatric patients presented with head trauma. After applying our inclusion/exclusion criteria, 1371 patients were included in the final analysis.

Inclusion criteria were the following:

- Age ≤ 18 years
- Presentation within 24 h of head trauma
- Glasgow Coma Scale (GCS) score ≥ 14

Exclusion criteria were the following:

- Penetrating trauma
- Known brain tumors or neurological disorders
- Presence of ventricular shunts
- Bleeding disorders or anticoagulation therapy
- Multiple trauma
- Transfer from other facilities
- Incomplete medical records
- Previous neuroimaging at other facilities

Of the initial 1892 patients:

- 287 were excluded due to presentation > 24 h after injury
- 98 were transferred from other facilities
- 76 had incomplete medical records
- 42 had multiple trauma
- 12 had bleeding disorders
- 6 had ventricular shunts

We collected baseline characteristics, the symptoms presented, the number of CT scans performed, and the patient’s outcomes in terms of ICI and NSI. Moreover, we analyzed the ED crowding, the ED length-of-stay (LOS), and the presence of ED boarding. All data were provided by the San Matteo Hospital Foundation database, which keeps files regarding all services provided by its ED.

An ad hoc query was performed to obtain the data of interest. Patients’ names were replaced with anonymous codes to ensure blinding of personal data.

The study protocol was approved by the Ethical Committee “Fondazione I.R.C.C.S. Policlinico San Matteo di Pavia” (Code 00478512/23). Patients provided informed consent for the use and processing of their data for medical and research purposes on admission to the ED.

### Endpoints

The primary objective of this study was to analyze the impact of the PECARN protocol on the number of head CT scans performed in the pediatric ED population admitted to a level 2 trauma center due to mild head injury. The secondary objective was to evaluate the impact of the PECARN protocol on the ED crowding, the ED LOS, and the ED boarding.

### PECARN TBI algorithm

The PECARN TBI algorithm is a clinical decision rule that aims to identify children at very low risk for clinically important TBI (Table 1). This validated pediatric algorithm predicts the likelihood of the above and guides the decision to perform a CT [17, 18].

**Table 1:** PECARN TBI algorithm

Age group	Criteria for low risk classification
< 2 years	• Normal mental status • Normal behavior (per caregiver) • No loss of consciousness • No serious injury mechanisms • No non-frontal scalp hematoma • No evidence of skull fracture
2–15 years	• Normal mental status • No loss of consciousness • No serious injury mechanisms • No vomiting • No severe headache • No signs of basilar skull fracture

If all the above criteria are present, the patient is deemed at low risk for a clinically significant intracranial finding

### Measurement of crowding

Several indices have been proposed to measure crowding [19, 20]. The most used indices can be grouped as follows:

- ◦ Input crowding indices: disease severity and complexity, waiting times.
- ◦ Throughput crowding indices: process time and LOS.
- ◦ Output crowding indices: the percentage of patients who experienced access block and boarding, and the total access block and boarding time.

"Waiting time” is defined as the time from the initial registration, entering the ED, to the time of medical consultation. LOS is the period from the arrival to the admission or discharge. Access block demarks as a LOS longer than 8-h from presentation to medical attendance [21, 22]. Total access block time represents the overall duration of access block for all patients studied [23]. Boarding is the time that passes from the decision to admit the patient and arrival to the hospital ward; some authors describe boarding as a LOS longer than 6-h to admission [24]. Thus, the total boarding time represents the aggregate duration of boarding for all patients studied [22, 24–27].

### Statistical analysis

Continuous variables were described with mean and standard deviation (SD) if normally distributed, with median and interquartile range if not normally distributed, and categorical variables were expressed as counts and percentages. Associations between categorical variables were studied using the Pearson *χ*^2^ test or the Fisher’s exact test. Continuous variables were compared between two groups using the Student’s *t* test or the analogous non parametrical test of Mann–Whitney.

Each variable was tested in a univariate logistic regression model to assess potential statistical significance in association with the CT results. The risk factors of interest with statistical significance at the univariate analysis and for which no collinearity was reported were included as regressors in a multivariate logistic model.

All tests were two-sided. The significance level was set at α = 0.05. A *p* value < 0.05 was considered statistically significant. Data analysis was performed using the STATA statistical package (version 15 or later; Stata Corporation, College Station, TX, USA).

## Results

### Baseline characteristics

A total of 1371 pediatric patients were consecutively admitted to our ED for TBI (Table 2).

**Table 2:** Baseline patient characteristics (*n* = 1371)

Variable	*n*	%
Age (< 2 years)	504	36
Sex (male)	818	59.75
*Transportation mode*		
Ambulance	87	6.36
Hospital ambulance	4	0.29
Public force	4	0.29
Autonomous	1162	84.88
Basic rescue	87	6.36
Advance rescue with doctor	15	1.10
Helicopter rescue	2	0.15
Intermediate rescue with nurse	7	0.51
*Triage acuity*		
Triage code–I	15	1.1
Triage code–II	102	7.4
Triage code–III	33	2.4
Triage code–IV	1219	88.9
Triage code–V	0	0
Major trauma	13	0.9
Main symptom complained		
Headache nausea or vomiting	1282	93.64
Syncope	3	0.22
Wounds	45	3.29
Major trauma	13	0.95
Dizziness	15	1.10
Syncope	4	0.29
Other signs and symptoms faded as general malaise	3	0.22
bleeding	1	0.07
Causal factors	n	%
Domestic	792	57.85
School	119	8.69
Other places	239	17,46
Violence	14	1.02
Road	88	6,43
Sport	87	6.36
Morbid state	29	2.12

After admission, all patients were assessed following the PECARN TBI algorithm. The majority of patients were male (59.8%) with a median age of 5 years. 36.8% of the patients were less than 2 years old and 63.2% were older than 2 years but less than 15 years old. Only 13 (1%) had criteria for major trauma with a GSC below 14. The main symptoms documented at admission were headache, often presented with nausea (1283 patients), wounds requiring suturing (46 patients), severe trauma (13 patients), neurological complaints (3 patients), dizziness (3 patients), syncope (3 patients), and the others had signs and symptoms such as general malaise.

Table 3 shows the diagnostic tests and specialist examinations performed on the included patients.

**Table 3:** Exams performed

Imaging required		
	*n*	%
Cranial CTs	4	0.29
Cervical CTs	1	0.07
Cerebral CTs	50	3.65
Massive facial CTs	4	0.29
Rx skull	28	2.05
Nasal bone X-ray	98	7.16
Cervical column X-ray	39	2.85
Specialist visits required		
	*n*	%
Visit neurosurgery	815	59.53
Eye examination	27	1.24
Maxillo facial visit	3	0.22
Pediatric surgery visit	80	5.84
ENT visit	143	10.45

The most frequent specialist examination performed was the neurosurgical consultation (approximately 59% of patients). The time required to perform exams and consultations after the request are shown in Table 4.

**Table 4:** Time elapsed from the examination request to the examination performance

Exam performed	TDE (min)
Imaging required	
Head CT	9.7
Cervical CT	2.0
Cerebral CT	21.1
Maxillo-facial CT	4.0
Rx skull	7.9
Nasal bone X-ray	4.9
Cervical column X-ray	15.8
Specialist visits required	
Neurosurgery consultation	23.0
Eye examination	17,.5
Maxillo-facial visit	2.3
Pediatric surgery visit	14.0
ENT visit	15.7

Data are reported as median (iqr); *TDE* time doc to exam

For every exam or consultation, this time was, on average, less than 30 min. Seven hundred ninety-nine patients arrived due to domestic injury, 119 children due to an injury at school, 239 children due to injury in other locations, 15 children attributed to violence from others, 88 children due to road accidents, 87 children due to sports accidents, and 29 children had trauma during a morbid state.

### Outcomes

Of the 1371 patients evaluated, only 59 (4.3%) underwent CT scanning based on PECARN criteria. Among these, seven patients (0.51%) were found to have intracranial bleeding, with only one patient (0.07%) requiring neurosurgical intervention. Importantly, among the 1312 patients who did not receive CT scans, none returned to our facility with delayed intracranial bleeding or neurological deterioration. The 1257 patients discharged home received standardized instructions for 28-h observation and warning signs requiring immediate return. Our follow-up data showed no cases of missed significant intracranial injuries among patients managed without CT scanning. This is particularly noteworthy as our facility is the only emergency department in the area with 24-h neurosurgical coverage and CT availability, making it unlikely that patients with delayed complications would seek care elsewhere. Among these, five cervical CTs and one maxillo-facial CT scans were obtained. Crowding indices in ED are shown in Table 5.

**Table 5:** Crowding indices

Input factors		
	Waiting time (min)	
	Mean (sd)	Median (iqr)
Triage code 4 and 5	40.57 (42.75)	25.97 (12.13; 53.5)
Triage code 3	35.15 (36.31)	23.58 (9.87; 47.1)
Triage code 2	27.63 (25.80)	19.36 (8.67; 42.8)
Triage code 1	4.55 (3.95)	3.9 (1.7; 6.33)
Throughput factors		
Time doc to exam (min)		
	Mean (sd)	Median (iqr)
Triage code 4 and 5	18.19 (31.66)	5.85 (2.15; 18.13)
Triage code 3	8.44 (8.94)	5.82 (2.58; 11.02)
Triage code 2	18.09 (22.75)	10.43 (3.27; 24.28)
Triage code 1	6.41 (8.22)	5.28 (0.87; 6.2)
Process time (min)		
	Mean (sd)	Median (iqr)
Triage code 4 and 5	109.81 (74.97)	94.32 (55.65; 144.05)
Triage code 3	128.65 (69.41)	113.65 (85.9; 160.37)
Triage code 2	176.77 (148.66)	143.87 (80.97; 221.15)
Triage code 1	152.54 (96.15)	145.33 (86.47; 227.2)
LOS time (min)		
	Mean (sd)	Median (iqr)
Triage code 4 and 5	150 (85.14)	135.15 (89.95; 189.35)
Triage code 3	163 (78.81)	166.48 (108.15; 210.25)
Triage code 2	204.40 (152.70)	67.69 (109.05; 256.2)
Triage code 1	154.78 (92.77)	147.83 (88.17; 239.67)
Output factors		
Boarding (*n* %)		4 (0.29%)
Exit block (*n* %)		0–(0%)
boarding total time (min)		291.05
time boarding, median [iqr] (min)		72.67 (53.87–91.66)
boarding minimum time (min)		37.28
boarding maximum time (min)		108.42

Continuous data are reported as mean (sd) or as median (iqr)

The univariate and multivariate analysis showed that older children (> 2 years old) were more likely to undergo CT (Table 6). The likelihood of undergoing CT increases significantly for more severe triage codes. Regarding the throughput factors, the mean process-time was 2 h, and 3 h for Triage-codes 1 and 2. The patients in need of observation were placed in the pediatric observation unit.

**Table 6:** Univariate logistic models result

	OR	95% CI	*p* value
Sex, M	1.37	0.80–2.33	0.254
Age (years)	1.33	1.26–1.41	< 0.001
Age ≥ 2 years	5.86	2.51–13.68	< 0.001
Triage code (baselevel Triage code IV)			
Triage code III	3.21	0.73–14.17	0.124
Triage code II	19.75	10.94–35.63	< 0.001
Triage code I	56.81	19.06–169.29	< 0.001
Waiting time	1.00	0.99–1.00	0.598
LOS	1.00	1.00–1.01	< 0.001
Time doc to exams	1.00	0.99–1.01	0.929
Process time	1.01	1.00–1.01	< 0.001
Multivariate logistic models result			
Model a)	OR	95% CI	*p* value
Age (years)	1.32	1.23–1.42	< 0.001
Triage code (baselevel Triage code IV)			
Triage code III	3.86	0.81–18.48	0.091
Triage code II	15.66	8.17–30.02	< 0.001
Triage code I	55.63	16.14–191.71	< 0.001
Model b)			
Age ≥ 2 years	4.73	1.96–11.39	0.001
Triage code (baselevel Triage code IV)			
Triage code III	3.18	0.71–14.24	0.1330
Triage code II	18.14	9.95–33.05	< 0.001
Triage code I	47.83	15.59–146.77	< 0.001

*LOS* Length of ED stay

Overall, the average ED-LOS was 155 min, 150 min for Triage-code- 4, 164 min for Triage-code- 3; 204 min for Triage-code- 2, and 154 for Triage-code- 1. No patients experienced access block and only 4 patients (0.3%) experienced boarding. In this case, the average boarding time was 72 min.

One thousand two hundred fifty-seven patients were discharged with a leaflet including instructions for observation at home for a further period of 28 h (Table 7). Seventy-three patients required hospitalization, 14 patients refused to be admitted, and 27 left before the end of the care process and signed a special form for the refusal of the observation.

**Table 7:** Outcomes of included patients

Outcomes	*N*	%
Dimission	1257	91.7
Hospitalized	72	5.3
Transferred	1	0.1
Left without being seen	27	2.0
Refuses hospitalization	14	1.0
Re-entries for bleeding	0	0
Intra-cranial bleeding	7	0.51
Need for neurosurgery	1	0.07

Only seven patients presented with bleeding, and one required an emergent surgical intervention. There were no re-entries for bleeding.

## Discussion

During the study period, children presenting with mild TBI were evaluated following our protocol. Emergency physicians promptly assessed these cases and ordered examinations and consultations based on clinical findings. The collaboration between emergency physicians and neurosurgeons supported protocol implementation. Following PECARN criteria, CT scans were performed in 4.3% of cases. Recent research has focused on developing evidence-based decision-making tools that incorporate clinical variables obtained from patient history and physical examination [28]. The goal has been to more accurately predict the risk of specific outcomes (such as intracranial lesions) and guide clinical decision-making through Clinical Decision Rules (CDRs) [29, 30]. While these predictive methods are heterogeneous and challenging to compare, the increasing use of brain CT has raised concerns about radiation exposure risks. Children are particularly vulnerable due to their tissues’ enhanced radiation sensitivity and longer life expectancy, during which radiation-induced oncogenic damage may manifest as increased rates of leukemia, brain tumors, and other solid tumors [31–37].

Brain CT is indicated in cases of major trauma where the risk of intracranial injury is high (> 20%). According to Hennelly et al., the optimal risk threshold justifying CT use is 4.8%. CT scanning is strongly recommended as the initial diagnostic test in children with a risk of 5% or higher, as well as in cases showing significant clinical deterioration. However, CT imaging is not indicated for children at lower risk levels. Studies have shown that 10% of skull radiographs and CT scans revealed skull fractures, with half of these cases requiring hospitalization. The PECARN score has emerged as the most reliable tool among existing scoring systems for determining CT scan necessity in infants with head trauma [38–40]. Our implementation of the PECARN TBI algorithm resulted in significantly lower CT scan utilization rates compared to previously published data [41–43].

Both univariate and multivariate analyses demonstrated that the protocol takes a conservative approach with younger patients (< 2 years old). As expected, patients assigned more urgent triage codes (1 and 2) were more likely to undergo CT scanning due to their more complex presentations and severe symptoms. This population also demonstrated a higher bleeding risk, further justifying the increased use of CT imaging. We sought to evaluate whether an observation-based protocol could help reduce hospital crowding [7–14, 19, 22, 24, 44, 45]. Implementation of the PECARN TBI algorithm did not increase ED crowding. Access times remained consistent with Italian ministerial guidelines and comparable to other institutions. Process times stayed within the 4–6-h window recommended by scientific societies [46, 47]. We observed very low boarding rates with no exit blocks. The dedicated observation unit effectively managed these patients while limiting crowding and reducing CT scan utilization. This well-structured pathway, with its dedicated spaces, enabled efficient patient management without increasing department congestion. Symptom assessment proved particularly challenging in children under 2 years of age, as their presentations were often vague and difficult to characterize. Pain and headache emerged as the most commonly reported symptoms. While obtaining reliable historical data is already challenging in adults presenting to the ED, it becomes even more difficult in pediatric trauma cases. Symptom identification is particularly challenging in children under 2 years of age, as their presentations tend to be vague and nonspecific. While pain and headache were the most frequently reported symptoms, obtaining reliable historical data in pediatric trauma cases proves even more challenging than in adults presenting to the ED [48]. Children often struggle to communicate their symptoms either due to young age or fear. Additionally, obtaining accurate information can be complicated when accompanying adults were not present during the injury, a situation particularly problematic with infants. In our study population, most mild head injuries occurred in domestic or recreational settings.

Neurosurgical consultation was requested in more than half of the cases, though radiological examinations were necessary in only a small portion. Our rates of hospitalization and surgical intervention were lower than those reported in other studies [28]. The absence of ED return visits to our facility, combined with these findings, suggests the algorithm’s high safety profile. As our hospital houses the city’s only Emergency Department with 24-h neurosurgical coverage and CT availability, this data on return visits, though retrospective, carries particular significance. While our multivariate logistic regression models effectively identified key predictors of CT utilization, we acknowledge certain limitations of this traditional statistical approach. More sophisticated machine learning techniques, such as random forests or neural networks, could potentially capture complex non-linear relationships between clinical variables and imaging needs. Future studies may benefit from exploring these advanced analytical methods to develop more precise prediction tools for identifying patients requiring neuroimaging. The remarkably low boarding rates (0.3%) and absence of exit block in our study warrant careful interpretation within the context of our hospital’s specific characteristics. As a level 2 trauma center with approximately 100,000 annual ED visits, we benefit from dedicated pediatric observation units and established protocols for rapid specialty consultation. Additionally, our 24-h neurosurgical coverage and immediate CT availability likely facilitated efficient patient flow. These institutional resources and operational features may not be universally available, potentially limiting the generalizability of our throughput metrics to centers with different staffing models or resource constraints. Future multi-center studies across diverse hospital settings would help validate whether similar operational efficiencies can be achieved in facilities with varying resource levels.

The higher number of visits to our center compared to previous studies, along with the notable percentage of patients leaving before treatment completion, may indicate a regional tendency to seek emergency care for pediatric head trauma. This pattern might be partly attributed to limited availability of rapid-access outpatient pediatric care in our area.

This study has several limitations. First, its retrospective design may have introduced selection bias and limited our ability to collect certain clinical data. Second, although our hospital is the only facility in the area with 24-h neurosurgical coverage and CT availability, we cannot completely rule out that some patients may have sought care at other facilities for subsequent complications. Third, the single-center nature of the study may limit the generalizability of our findings to other healthcare settings with different resources or patient populations. Additionally, the lack of a control group prevents direct comparison of outcomes between the PECARN algorithm and other clinical decision rules. Finally, while we tracked ED return visits, our follow-up period was limited, and we may have missed longer-term outcomes or complications. While our data demonstrates excellent short-term safety with no returns for bleeding complications, we cannot definitively comment on potential subtle neurocognitive or behavioral changes that might manifest weeks to months after the initial injury. This limitation is especially relevant for mild TBI in children, where symptoms may evolve gradually and affect developmental trajectories. Future prospective studies implementing scheduled follow-up assessments at regular intervals (e.g., 2 weeks, 1 month, 3 months) would be valuable to evaluate both the immediate and long-term safety of PECARN-guided CT utilization strategies. Such studies should include age-appropriate neurocognitive testing and standardized symptom assessment to detect any delayed manifestations of injury that could be missed in the acute setting.

## Conclusion

The implementation of the PECARN algorithm in our ED resulted in a significant reduction in CT scan utilization for pediatric head trauma without compromising patient safety or outcomes. The protocol proved particularly effective when combined with a dedicated observation unit, which helped manage patients efficiently while preventing ED crowding. Our experience demonstrates that this evidence-based approach can successfully balance the need to identify clinically significant head injuries with the imperative to minimize radiation exposure in pediatric patients. These findings support the broader adoption of the PECARN algorithm in similar emergency care settings, particularly when accompanied by appropriate observation protocols and resources.

## Data Availability

The datasets used and/or analysed during the current study are available from the corresponding author on reasonable request.
